# Secure Fusion with Labeled Multi-Bernoulli Filter for Multisensor Multitarget Tracking Against False Data Injection Attacks

**DOI:** 10.3390/s25113526

**Published:** 2025-06-03

**Authors:** Yihua Yu, Yuan Liang

**Affiliations:** 1 School of Mathematical Science, Beijing University of Posts and Telecommunications, Beijing 100876, China; yuyihua@bupt.edu.cn; 2 School of Economics, Beijing Institute of Technology, Beijing 100081, China

**Keywords:** false data injection (FDI) attack, information fusion, Kullback–Leibler divergence (KLD), labeled multi-Bernoulli (LMB) filter, multitarget tracking

## Abstract

**Highlights:**

**What are the main findings?**

- A multisensor multitarget tracking algorithm against the false data injection (FDI) attacks over networks.
- A detection method for FDI attacks based on Kullback–Leibler divergence (KLD) between labeled multi-Bernoulli densities.

**What is the implication of the main finding?**

- The proposed algorithm can efficiently detect/defend the FDI attacks and provide reliable tracking performance.

**Abstract:**

This paper addresses multisensor multitarget tracking where the sensor network can potentially be compromised by false data injection (FDI) attacks. The existence of the targets is not known and time-varying. A tracking algorithm is proposed that can detect the possible FDI attacks over the networks. First, a local estimate is generated from the measurements of each sensor based on the labeled multi-Bernoulli (LMB) filter. Then, a detection method for FDI attacks is derived based on the Kullback–Leibler divergence (KLD) between LMB random finite set (RFS) densities. The statistical characteristics of the KLD are analyzed when the measurements are secure or compromised by FDI attacks, from which the value of the threshold is selected. Finally, the global estimate is obtained by minimizing the weighted sum of the information gains from all secure local estimates to itself. A set of suitable weight parameters is selected for the information fusion of LMB densities. An efficient Gaussian implementation of the proposed algorithm is also presented for the linear Gaussian state evolution and measurement model. Experimental results illustrate that the proposed algorithm can provide reliable tracking performance against the FDI attacks.

## 1. Introduction

Multitarget tracking is to jointly detect and track the number of targets and their individual trajectories of multiple mobile targets. The number of targets is time-varying, i.e., new targets can appear and existing targets can disappear. The sensor’s measurements suffer from missed detection, false alarms, and uncertain associations between targets and measurements. Multitarget tracking is a fundamental topic in many engineering fields, including traffic control [1], remote sensing [2], and computer vision [3,4].

Various methods have been developed to perform the multitarget tracking. A popular class is the random finite set (RFS)-based recursive Bayesian filtering methods, which are mainly composed of two types: unlabeled and labeled methods. The unlabeled RFS-based methods include the probability hypothesis density (PHD) filter [5], the cardinalized PHD (CPHD) filter [6], and the multi-Bernoulli (MB) filter [7]. Compared to the unlabeled methods, the labeled RFS-based methods not only track the target states but also incorporate the target labels into the target states. The labeled RFS-based methods include the generalized labeled multi-Bernoulli (GLMB) filter [8] and the labeled multi-Bernoulli (LMB) filter [9,10].

The availability of low-cost sensors has employed multiple sensors to cooperatively perform the tracking tasks. A local estimate is first computed with the measurements of each sensor. Then, the global estimate is generated by fusing all local estimates. The most commonly used fusion method is the generalized covariance intersection (GCI) fusion [11], also called as exponential mixture density [12]. The global estimate produced by GCI fusion is actually the one that minimized a weighted average of Kullback–Leibler divergence (KLD) from all local estimates to itself [13,14]. It can be interpreted as the one that leads to minimum cross-entropy from the information-theoretic viewpoint. Using the GCI fusion rule, many algorithms have been proposed for different multisensor multitarget tracking scenarios, e.g., the sensors have different fields of view [15,16], sensor fusion with event-triggered communication [17,18,19], and so on. For the event-triggered communication in [17,18,19], each sensor evaluates the KLD between the current local density and the last transmitted one and selectively sends the information that is worth transmitting, i.e., the KLD exceeds a threshold.

During these works, it is assumed that the sensor networks are secure and trusted all the time. Unfortunately, the sensor networks, such as the networked radar system [20] and the intelligent transportation networks [21], are vulnerable to unforeseen breaches in security. Due to the open communication between sensors, the data may not be transmitted with proper security protections. It is of great importance to accurately and quickly detect the adversarial attacks.

Among various types of adversarial attacks, false data injection (FDI) attacks [22,23] are a major concern. The FDI attacks are known as specific deception attacks or integrity attacks. With a successful FDI attack, an adversary can gain access to the communication and modify the transmitted data between sensors, which can manipulate the estimated values of the state variables and disrupt the system’s operation.

How to detect and defend against FDI attacks has attracted much research attention. For single-target tracking in [24], the KLDs between local posteriors are used to partition the sensors into secure and insecure sensors. In [25], the relative entropy was utilized as a stealthiness metric to detect whether the data transmitted through the wireless network are attacked. In [26], the authors investigate the security of distributed filtering under false data injection attacks, and a new protection strategy is proposed. In [27], the authors investigate a distributed secure estimation problem for a nonlinear stochastic system subject to a false data injection attack. In [28], the authors apply sequential attack strategies to wireless mesh networks and conduct a comparative analysis with synchronous strategies. In these studies, the existence of the system state is known and time-invariant, so the dimension of system state is known and time-invariant. This is different from the multitarget tracking problem, where the targets may appear or disappear, i.e., the existence of system state is not known and time-varying, so the dimension of the system state is not known and time-varying. It is necessary to design the FDI detection method that is suitable for the multitarget tracking.

In this paper, we address multisensor multitarget tracking following a Bayesian filtering perspective where the sensor network may be compromised by FDI attacks. The transmitted data between sensors may be modified by the adversary. We propose a multisensor, multitarget tracking algorithm that can detect the possible FDI attacks over the network and provide robust and accurate tracking performance. More specifically, a local estimate is first generated from the measurements of each sensor based on the LMB filter. Then, a detection method for FDI attacks is derived with an information-theoretic criterion, where the KLD between LMB densities is utilized as a metric to quantify the similarity/difference of two local estimates. If the KLD between a local estimate and the reference estimate exceeds a given threshold, the local estimate is decided to be compromised by FDI attacks. Finally, the global estimate is obtained by minimizing the weighted sum of information gains from all secure local estimates to itself. An efficient Gaussian implementation of the proposed algorithm is also presented for the linear Gaussian state evolution and measurement model.

The contributions of this paper can be summarized as follows: (1) a detection method for FDI attacks is derived based on the KLD between LMB densities, and the statistical characteristics of the KLD are analyzed when the measurements are secure or compromised by FDI attacks, from which the value of the threshold is selected; (2) a set of suitable weight parameters are selected for the information fusion of LMB densities.

The rest of this paper is organized as follows. Section 2 describes the system model and the FDI attacks for multisensor multitarget tracking. Section 3 presents the proposed multisensor multitarget tracking algorithm that can detect and defend against the FDI attacks. Extensive numerical experiments are provided in Section 4. The paper is concluded in Section 5.

## 2. Problem Formulation and Background

In this section, we describe the state evolution model, measurement model, and FDI attacks for the multisensor multitarget tracking problem. This section also briefly recalls the labeled RFS and LMB filters.

### 2.1. Target State and Evolution Model

Suppose that there are multiple targets in the surveillance area at the time k. The state vector of each target is defined as $x_{k}=\left(p_{x,k},v_{x,k},p_{y,k},v_{y,k}\right)$, where $\left(p_{x,k},p_{y,k}\right)$ denotes the position and $\left(v_{x,k},v_{y,k}\right)$ denotes the velocity of the target. The state could be extended to incorporate more variables if it is required. Both the number of targets and the state of each target are not known and time-variant.

Each target at a time k−1 may continue to exist or disappear at the next time k. We denote the surviving probability that the target with state $x_{k-1}$ will continue to exist at the next time as $p_{s}(x_{k-1})$. If the target $x_{k-1}$ continues to exist, its state evolves according to the nearly constant velocity model [29] as

(1) $$x_{k}=A_{k}x_{k-1}+u_{k},$$

where the transition matrix $A_{k}$ is given by

(2) $$A_{k}=I_{2}⊗\left(\begin{array}{cc} 1 & T\\ 0 & 1 \end{array}\right),$$

with the _*n* × *n*_ identity matrix $I_{n}$, the time interval T, and the Kronecker product ⊗. The term $u_{k}$ is the additive Gaussian process noise with zero mean and covariance.

(3) $$Q_{k}=q_{u}I_{2}⊗\left(\begin{array}{cc} T^{3}/2 & T^{2}/2\\ T^{2}/2 & T \end{array}\right).$$

with the process noise intensity $q_{u}$. Accordingly, the single-target transition density is given by

(4) $$f(x_{k}|x_{k-1})∼N\left(x_{k};A_{k}x_{k-1},Q_{k}\right).$$

Other motion models can also be used in the state space framework. The multiple targets move independently in the surveillance area.

In addition, a set of new targets are born in the surveillance area at each time step. The number of new targets is distributed according to a discrete probability distribution, and the state of each new target is distributed according to a spatial density over the surveillance area.

### 2.2. Sensor Network and Measurement Model

We consider a set of N sensors V≜{1,⋯,N}, which are geographically dispersed in the surveillance area. Each sensor has sensing, communication, and processing capabilities.

For each target $x_{k}$ at time k, the _*n*_th sensor detects the target with probability $p_{d}(x_{k})$, so the probability of the missed detection is $q_{d}(x_{k})=1-p_{d}(x_{k})$. If the target $x_{k}$ is detected by the _*n*_th sensor, the measurement is governed by the single-target measurement model.

(5) $$y_{n,k}=B_{n,k}x_{k}+v_{n,k},$$

where the measurement matrix is $B_{n,k}=\left(\begin{array}{l} \begin{array}{cccc} 1 & 0 & 0 & 0 \end{array}\\ \begin{array}{cccc} 0 & 0 & 1 & 0 \end{array} \end{array}\right)$, and $v_{n,k}$ is the Gaussian measurement noise with zero mean and covariance $R_{n,k}$. Other measurement models can also be used in the state space framework.

Moreover, the _*n*_th sensor may detect a set of false alarms in the surveillance area. It is usually assumed that the number of false alarms is distributed according to a discrete Poisson distribution, and each false alarm is generated according to a spatial density κ(⋅) over the surveillance area.

At each time k, the sensors $n\in \left\{2,\cdots ,N\right\}$ transmit their measurements to the first sensor. The first sensor recursively estimates the multitarget states based on the measurements from all sensors up to time k.

### 2.3. FDI Attacks

The sensors $n\in \left\{2,\cdots ,N\right\}$ transmit their measurements to the first sensor through some wired or wireless transmission. Generally, the sensors, such as the networked radar system [20] and the intelligent transportation networks [21], are spatially deployed in a monitoring region. The data may not be transmitted with proper security protections because of the broadcast nature. The wired or wireless connections between the _*n*_th sensor and the first sensor can be maliciously compromised.

If one measurement $y_{n,k}$ from the _*n*_th sensor arrives at the first sensor, the received measurement may be compromised by FDI attacks. The attacker hijacks the data packets from the communication channel and injects data packets that are intentionally inaccurate [20]. The compromised measurement under the FDI attack is modified as [20].

(6) $$z_{n,k}=y_{n,k}+\vartheta_{n,k},$$

where $\vartheta_{n,k}$ is the FDI data injected by the attacker. A successful FDI attack can cause the state estimation to generate erroneous values. Since the FDI attack does not undermine the integrity of the data, it is stealthy (e.g., fools the popular $\chi^{2}$ detector) and difficult to be detected. It is important to protect the security of the transmitted data and detect/defend the FDI attacks in the networks.

### 2.4. Labeled RFS and LMB Filter

In order to distinguish individual targets, a distinct label l∈L is augmented to each target x∈Ω [8], where Ω denotes the single-target state space. The target with a label is denoted as $x=\left(x,l\right)$. Let f:Ω×L→L be the projection $f\left(\left(x,l\right)\right)=l$. The space of all labels for targets born at time k is denoted by $L_{k}$. In the RFS formulation, the state of multiple targets with labels at time k can be modeled as a labeled RFS.

(7) $$X_{k}=\left\{x_{k}^{1},\cdots ,x_{k}^{\left|X_{k}\right|}\right\},$$

where each target $x_{k}^{i}(i=1,\cdots ,|X_{k}|)$ takes values in the state space Ω×L. Then, $f\left(X\right)=\left\{f\left(x\right):x\in X\right\}$ is the set of labels of the RFS X. Each element in X has distinct label, i.e., $\left|f\left(X\right)\right|=\left|X\right|$ (the sets $f\left(X\right)$ and X have the same cardinality). Similarly, the measurement set from the _*n*_th sensor can also be described as a RFS

(8) $$Z_{n,k}=\left\{z_{n,k}^{1},\cdots ,z_{n,k}^{\left|Z_{n,k}\right|}\right\},$$

where each measurement $z_{n,k}^{j}(j=1,\cdots ,|Z_{n,k}|)$ takes values in a single-target measurement space Ξ.

An LMB density can be completely specified by the parameter set [9].

(9) $$\pi ={\left\{\left(r^{\left(l\right)},p^{\left(l\right)}\right)\right\}}_{l\in L},$$

where $r^{\left(l\right)}$ is the existence probability of the target with label l, and $p^{\left(l\right)}(\cdot )$ is its spatial density. The multitarget density of LMB RFS with the parameter set π is given by [9].

(10) $$\pi \left(X\right)=\delta_{\left|X\right|}\left(\left|f\left(X\right)\right|\right)\omega \left(f\left(X\right)\right)p^{X},$$

where $\delta_{\left|X\right|}\left(\left|f\left(X\right)\right|\right)=1$ if $\left|X\right|=\left|f\left(X\right)\right|$ and 0 otherwise, which is the distinct label indicator, and

(11) $$\omega \left(L\right)=\prod_{i\in L}(1-r^{\left(i\right)})\prod_{l\in L}\frac{r^{\left(l\right)}}{1-r^{\left(l\right)}},$$

(12) $$p^{X}=\prod_{(x,l)\in X}p^{\left(l\right)}(x),$$

in which L is a set of labels.

The single-sensor LMB filter estimates the multitarget states using the measurements from one sensor. For notation simplicity, we temporarily remove the index (n,k) of $Z_{n,k}$ in (8), and use the notation Z to denote the measurements from any one sensor.

At times k, we denote the LMB prior density by

(13) $$\pi_{k-1}={\left\{\left(r_{k-1}^{\left(l\right)},p_{k-1}^{\left(l\right)}\right)\right\}}_{l\in L_{-}},$$

The newly born targets at time k is also modeled as an LMB density.

(14) $$\pi_{b}={\left\{\left(r_{b}^{\left(l\right)},p_{b}^{\left(l\right)}\right)\right\}}_{l\in L_{k}},$$

Under the Bayes framework, the LMB filter recursively estimates the multitarget states with the LMB densities, including two steps: prediction and update.

The predicted multi-target density is an LMB density with the parameters [9].

(15) $${\left\{\left(r_{k|k-1}^{\left(l\right)},p_{k|k-1}^{\left(l\right)}\right)\right\}}_{l\in L}={\left\{\left(r_{S,k|k-1}^{\left(l\right)},p_{S,k|k-1}^{\left(l\right)}\right)\right\}}_{l\in L_{-}}\cup {\left\{\left(r_{b}^{\left(l\right)},p_{b}^{\left(l\right)}\right)\right\}}_{l\in L_{k}},$$

where $L=L_{-}\cup L_{k}$, and

(16) $$r_{S,k|k-1}^{\left(l\right)}=\eta_{S}^{\left(l\right)}r_{k-1}^{\left(l\right)},$$

(17) $$p_{S,k|k-1}^{\left(l\right)}=\frac{\langle p_{S}^{\left(l\right)}(\cdot )f^{\left(l\right)}\left(x|\cdot \right),p_{k-1}^{\left(l\right)}(\cdot )\rangle }{\eta_{s}^{\left(l\right)}},$$

(18) $$\eta_{S}^{\left(l\right)}=\langle p_{S}^{\left(l\right)}(\cdot ),p_{k-1}^{\left(l\right)}(\cdot )\rangle ,$$

where $p_{S}^{\left(l\right)}(\cdot )=p_{s}(x,l)$ is the surviving probability and $f^{\left(l\right)}\left(x|\cdot \right)=f(x,l|\cdot ,l)$ is the transition probability of the labeled target (x,l), and the inner product notation is defined as $\langle f,g\rangle =\int f(x)g(x)dx$.

With the measurement Z from any one sensor, the updated multitarget density is approximated as an LMB density with the parameters [9].

(19) $$r_{k}^{\left(l\right)}=\sum_{(I,\theta )\in F(L)\times \Theta }1_{I}(l)\omega^{\left(I,\theta \right)}(Z),$$

(20) $$p_{k}^{\left(l\right)}(x)=\frac{1}{r_{k}^{\left(l\right)}}\sum_{(I,\theta )\in F(L)\times \Theta }1_{I}(l)\omega^{\left(I,\theta \right)}(Z)p^{\theta }(x,l),$$

where

(21) $$\omega^{\left(I,\theta \right)}(Z)=\omega_{k|k-1}^{\left(I\right)}{\left[\eta_{Z}^{\theta }\right]}^{I},$$

(22) $$p^{\left(\theta \right)}(x,l)=\frac{p_{k|k-1}^{\left(l\right)}(x)\varphi_{Z}(x,l;\theta )}{\eta_{Z}^{\left(\theta \right)}(l)},$$

(23) $$\eta_{Z}^{\left(\theta \right)}(l)=\langle p_{k|k-1}^{\left(l\right)}(\cdot ),\varphi_{Z}(\cdot ,l;\theta )\rangle ,$$

(24) $$\varphi_{Z}(x,l;\theta )=\left\{\begin{array}{l} \frac{p_{d}(x,l)g(z^{\theta (l)}|x,l)}{\kappa (z^{\theta (l)})},\;\;\mathrm{if}\;\;\theta (l)>0\\ 1-p_{d}(x,l),\;\;\;\;\;\;\;\mathrm{if}\;\;\theta (l)=0, \end{array}\right.$$

and Θ is the space of mapping I→{0,1,⋯,|Z|} such that θ(i)=θ(j)>0 if and only if i=j (whose elements represent track-to-measurement association hypotheses) F(L) denotes the set of all subsets of L, $1_{I}(l)=1$ if l∈I and 0 otherwise, $p_{d}(x,l)$ is the detection probability, and g(z|x,l) is the single-target likelihood function of the measurement z corresponding to the labeled target (x,l).

## 3. LMB-Based Multitarget Tracking Against FDI Attacks

We derive a detection method for FDI attacks based on the KLD between LMB densities. The secure local estimates are then fused into a global estimate.

### 3.1. Basic Construction

The sensors $n\in \left\{2,\cdots ,N\right\}$ transmit their measurements to the first sensor, then the first sensor estimates the multitarget states based on the measurements of all sensors. The estimation process includes three steps: (1) the first sensor computes a local estimate based on each sensor’s measurements with the LMB filter; (2) the first sensor detects the local estimates that are comprised by FDI attacks; (3) the first sensor fuses all secure local estimates into a global estimate.

At time k−1, we denote the LMB prior density at the first sensor as that in (13). With the prior density and the LMB born density in (14), the first sensor computes the local estimate from the _*n*_th sensor’s measurements, and we denote the parameter set as

(25) $$\pi_{n,k}={\left\{\left(r_{n,k}^{\left(l\right)},p_{n,k}^{\left(l\right)}\right)\right\}}_{l\in L}.$$

The corresponding LMB density with the form in (10) is denoted as $\pi_{n,k}$. Since all local estimates are computed from the same prior density and born density, the label set L is the same for all local estimates, and the labels corresponding to the same target in different set $\pi_{n,k}$ are also the same, i.e., there is no label mismatching problem.

Since the wired or wireless connections between the _*n*_th sensor and the first sensor may be maliciously compromised by FDI attacks, the compromised measurements will deteriorate the local estimates. It is necessary to detect the attacked sensor as well as the corresponding local estimates before the fusion process. In particular, since the first sensor directly has its measurements, it is not necessary to be transmitted over the wired or wireless connections, so it is assumed that the measurements of the first sensor are secure all the time.

For notation simplicity, we remove the index k in $\pi_{n,k}$ and $\pi_{n,k}$ because all operations are executed at time k in the rest of this section.

### 3.2. KLD-Based Detection of FDI Attacks

Because the measurements from the first sensor cannot be compromised by FDI attacks all the time, the local LMB density $\pi_{1}$ will be secure all the time. We consider $\pi_{1}$ as the reference density and utilize some metric for the discrepancy between each density $\pi_{n}$ ($n\in \left\{2,\cdots ,N\right\}$) and the reference density $\pi_{1}$ to detect the possible FDI attacks. We introduce a binary variable $b_{n}$ for the _*n*_th sensor such that $b_{n}=1$ if the measurements from the _*n*_th sensor are compromised by FDI attacks, and $b_{n}=0$ otherwise.

There are many ways to quantify the discrepancy of two densities. The KLD is a competitive alternative in many probabilistic detection problems. The KLD of two probabilistic densities p(x) and q(x) is defined as

(26) $$D_{KL}\left(p||q\right)=\int p(x)\ln\frac{p(x)}{q(x)}dx,$$

where $D_{KL}\left(p||q\right)\geq 0$, and $D_{KL}\left(p||q\right)=0$ if and only if p(x)=q(x). The KLD quantifies how close a density is to a reference density. The KLD admits an elegant interpretation from an information-theoretic viewpoint, i.e., it represents the information gain achieved when the density q is revised into the density p [30].

The concept of the KLD can be extended to the multitarget RFS density with the set integral. If p and q are two RFS densities, the discrepancy between p and q can be measured by the KLD.

(27) $$D_{KL}\left(p||q\right)=\int p(X)\ln\frac{p(X)}{q(X)}\delta X,$$

where the integral ∫⋅δX, is a set integral defined by

(28) $$\int f(X)\delta X=f(\emptyset )+\sum_{n=1}^{\infty }\frac{1}{n!}\int f(\{x_{1},\cdots ,x_{n}\})dx_{1}\cdots dx_{n}.$$

The KLD is not symmetric, i.e., $D_{KL}\left(p||q\right)\neq D_{KL}\left(q||p\right),$ in general. The *J divergence* [31] symmetrizes the KLD as

(29) $$J\left(p,q\right)=\frac{1}{2}\left(D_{KL}\left(p||q\right)+D_{KL}\left(q||p\right)\right)$$

where $D_{KL}\left(p||q\right)$ is usually called forward KLD and $D_{KL}\left(q||p\right)$ is reverse KLD [32].

By considering the discrepancy metric in (29), the following detection method for FDI attacks is adopted as

(30) $$b_{n}=\left\{\begin{array}{l} 1,\;\mathrm{if}\;J(\pi_{n},\pi_{1})>\tau \;\\ 0,\;\;\;\;\;\;\mathrm{otherwise}\; \end{array}\right.,$$

where the scalar _*τ*_ is a design parameter that is tuned so as to achieve a desired performance. We will discuss the choice of _*τ*_ in Section 4.2.

Substituting the LMB density $\pi_{n}$ with the parameter set $\pi_{n}={\left\{\left(r_{n}^{\left(l\right)},p_{n}^{\left(l\right)}\right)\right\}}_{l\in L}$ into the KLD (27) and using the definition of set integral, it can be proved that [33]

(31) $$D_{KL}(\pi_{n}||\pi_{1})=\sum_{l\in L}D_{KL}(r_{n}^{\left(l\right)}||r_{1}^{\left(l\right)})+\sum_{l\in L}r_{n}^{\left(l\right)}D_{KL}(p_{n}^{\left(l\right)}||p_{1}^{\left(l\right)}),$$

where

(32) $$D_{KL}(r_{n}^{\left(l\right)}||r_{1}^{\left(l\right)})=r_{n}^{\left(l\right)}\ln\frac{r_{n}^{\left(l\right)}}{r_{1}^{\left(l\right)}}+(1-r_{n}^{\left(l\right)})\ln\frac{1-r_{n}^{\left(l\right)}}{1-r_{1}^{\left(l\right)}},$$

(33) $$D_{KL}(p_{n}^{\left(l\right)}||p_{1}^{\left(l\right)})=\int p_{n}^{\left(l\right)}(x)\ln\frac{p_{n}^{\left(l\right)}(x)}{p_{1}^{\left(l\right)}(x)}dx.$$

Because all local estimates are computed from the same prior density and born density, there is no label mismatching problem, i.e., the term with label l in the local density $\pi_{n}={\left\{\left(r_{n}^{\left(l\right)},p_{n}^{\left(l\right)}\right)\right\}}_{l\in L}$ describes the same target as the term with label l in the local density $\pi_{1}={\left\{\left(r_{1}^{\left(l\right)},p_{1}^{\left(l\right)}\right)\right\}}_{l\in L}$. The KLD $D_{KL}(\pi_{n}||\pi_{1})$ can be computed in a label-wise manner.

### 3.3. Information Fusion

We denote the index set of secure local estimates after the detection of FDI attacks as

(34) $$U=\{n\in V:b_{n}=0\},$$

The local estimates $\pi_{n}$ in U are not considered to be compromised by FDI attacks. In order to combine all local estimates in U into a global estimate, the first sensor carries out the information fusion step. The goal of the fusion step is to compute the global density that encapsulates all the information provided by the local densities $\pi_{n}$ in U which can provide better estimation performance.

From a view of information theory, the fusion of the local densities $\pi_{n}$ in U is to find the density that has minimum divergence gain from all densities $\pi_{n}$ in U with respect to a suitable metric. The global density can be obtained by minimizing the weighted sum of the information gains from all local densities in U to itself as [13].

(35) $$\pi =\arg\underset{\pi }{\inf}\sum_{n\in U}w_{n}D(\pi ,\pi_{n}),$$

where $D(\pi ,\pi_{n})$ denotes the information gain from the density $\pi_{n}$ to the density π, and the non-negative weights $w_{n}$ reflect the relative information confidence of $\pi_{n}$ in comparison with the rest terms, which satisfies $\sum_{n\in U}w_{n}=1$. The KLD $D_{KL}(\pi_{n}||\pi )$ between $\pi_{n}$ and π is one of the popularly adopted metrics for the information gain.

It can be shown that the fusion density computed by the rule (35) with the KLD is given by [34].

(36) $$\pi (X)=\frac{{\prod_{n\in U}\left[\pi_{n}(X)\right]}^{w_{n}}}{\int {\prod_{n\in U}\left[\pi_{n}(X)\right]}^{w_{n}}\delta X}.$$

The rule in (36) is also called the GCI fusion rule. It provides a weighted geometric mean in the form of an exponential mixture of the local densities.

Because all local densities $\pi_{n}$ in U are LMB densities with the parameters $\pi_{n}={\left\{\left(r_{n}^{\left(l\right)},p_{n}^{\left(l\right)}\right)\right\}}_{l\in L}$, one can check that the global density π in (35) is also an LMB density with the parameters [33].

(37) $$r^{\left(l\right)}=\frac{\int \prod_{n\in U}{\left[r_{n}^{\left(l\right)}p_{n}^{\left(l\right)}(x)\right]}^{w_{n}}dx}{\prod_{n\in U}{\left(1-r_{n}^{\left(l\right)}\right)}^{w_{n}}+\int \prod_{n\in U}{\left[r_{n}^{\left(l\right)}p_{n}^{\left(l\right)}(x)\right]}^{w_{n}}dx},$$

(38) $$p^{\left(l\right)}(x)=\frac{\prod_{n\in U}{\left[p_{n}^{\left(l\right)}(x)\right]}^{w_{n}}}{\int \prod_{n\in U}{\left[p_{n}^{\left(l\right)}(x)\right]}^{w_{n}}dx}.$$

Since the same target is described with the same label in the different parameter sets ${\left\{\left(r_{n}^{\left(l\right)},p_{n}^{\left(l\right)}\right)\right\}}_{l\in L}$, the fusion step can be performed in a label-wise manner.

### 3.4. Implementation Issues

Each term of the LMB density in (14) of the newly born targets is commonly modeled as a Gaussian density.

(39) $$p_{b}^{\left(l\right)}(x)=N(x;\mu_{b}^{\left(l\right)},\Sigma_{b}^{\left(l\right)}).$$

where the model parameters $\mu_{b}^{\left(l\right)}$ and $\Sigma_{b}^{\left(l\right)}$ indicate the highest local concentrations of spontaneous birth, which can represent, for example, the airports where the target is mostly likely to appear [35].

The computation of $D_{KL}(r_{n}^{\left(l\right)}||r_{1}^{\left(l\right)})$ in (32) is direct. In order to develop a computationally feasible implementation of $D_{KL}(p_{n}^{\left(l\right)}||p_{1}^{\left(l\right)})$ in (33), we approximate the spatial density $p_{n}^{\left(l\right)}(x)$ as a Gaussian density.

(40) $$p_{n}^{\left(l\right)}(x)=N(x;\mu_{n}^{\left(l\right)},\Sigma_{n}^{\left(l\right)}).$$

This result can be obtained by the commonly used Gaussian mixture implementation of the LMB filter and suitable pruning/merging operations. Because each term $\left(r_{n}^{\left(l\right)},p_{n}^{\left(l\right)}\right)$ in $\pi_{n}$ describes only one target with the existence probability $r_{n}^{\left(l\right)}$ and the spatial density $p_{n}^{\left(l\right)}(x)$, the Gaussian approximation has enough accuracy.

Substituting the Gaussian form of $p_{n}^{\left(l\right)}(x)$ into (33), it will have

(41) $$\begin{array}{l} D_{KL}(p_{n}^{\left(l\right)}||p_{1}^{\left(l\right)})=\frac{1}{2}\left[\mathrm{Trace}\left({\left(\Sigma_{1}^{\left(l\right)}\right)}^{-1}\Sigma_{n}^{\left(l\right)}\right)-M+\ln\frac{\Sigma_{1}^{\left(l\right)}}{\Sigma_{n}^{\left(l\right)}}\right]\\ \;+{\left(\mu_{1}^{\left(l\right)}-\mu_{n}^{\left(l\right)}\right)}^{T}{\left(\Sigma_{1}^{\left(l\right)}\right)}^{-1}(\mu_{1}^{\left(l\right)}-\mu_{n}^{\left(l\right)}), \end{array}$$

where M denotes the dimension of the variable x, and $\mathrm{Trace}\left(\Sigma \right)$ means the trace of the matrix Σ.

Moreover, substituting the Gaussian form of $p_{n}^{\left(l\right)}(x)$ into (38), it will have

(42) $$p^{\left(l\right)}(x)=N(x;\mu^{\left(l\right)},\Sigma^{\left(l\right)}),$$

(43) $$\Sigma^{\left(l\right)}={\left(\sum_{n\in U}w_{n}{\left(\Sigma_{n}^{\left(l\right)}\right)}^{-1}\right)}^{-1},$$

(44) $$\mu^{\left(l\right)}=\Sigma^{\left(l\right)}\sum_{n\in U}w_{n}{\left(\Sigma_{n}^{\left(l\right)}\right)}^{-1}\mu_{n}^{\left(l\right)},$$

and $\int \prod_{n\in U}{\left[p_{n}^{\left(l\right)}(x)\right]}^{w_{n}}dx$ in (37) and (38) has the form

(45) $$\begin{array}{l} \int \prod_{n\in U}{\left[p_{n}^{\left(l\right)}(x)\right]}^{w_{n}}dx=\frac{\det{\left(2\pi \Sigma^{\left(l\right)}\right)}^{\frac{1}{2}}}{\sum_{n\in U}\det{\left(2\pi \Sigma_{n}^{\left(l\right)}\right)}^{\frac{w_{n}}{2}}}\\ \;\times \exp\left\{-\frac{1}{2}\left[\prod_{n\in U}\mu_{n}^{\left(l\right)}{\left(\Sigma_{n}^{\left(l\right)}\right)}^{-1}\mu_{n}^{\left(l\right)}-\mu^{\left(l\right)}{\left(\Sigma^{\left(l\right)}\right)}^{-1}\mu^{\left(l\right)}\right]\right\}. \end{array}$$

If the _*n*_ sensor is not compromised by FDI attacks, the value of $r_{n}^{\left(l\right)}$ will be closely approximate to the value of $r_{1}^{\left(l\right)}$, both of which are the existence probability of the same target with label l. Similarly, the value of $\Sigma_{n}^{\left(l\right)}$ is also closely approximate to $\Sigma_{1}^{\left(l\right)}$ with the assumptions of the shared prior and the uniform Kalman gains. So the values of $D_{KL}(r_{n}^{\left(l\right)}||r_{1}^{\left(l\right)})$ in (32) and $\mathrm{Trace}\left({\left(\Sigma_{1}^{\left(l\right)}\right)}^{-1}\Sigma_{n}^{\left(l\right)}\right)-M+\ln\frac{\Sigma_{1}^{\left(l\right)}}{\Sigma_{n}^{\left(l\right)}}$ in (41) are much smaller than the value of ${\left(\mu_{1}^{\left(l\right)}-\mu_{n}^{\left(l\right)}\right)}^{T}{\left(\Sigma_{1}^{\left(l\right)}\right)}^{-1}(\mu_{1}^{\left(l\right)}-\mu_{n}^{\left(l\right)})$ in (41). The main part of $D_{KL}(\pi_{n}||\pi_{1})$ will be decided by the quadratic form ${\left(\mu_{1}^{\left(l\right)}-\mu_{n}^{\left(l\right)}\right)}^{T}{\left(\Sigma_{1}^{\left(l\right)}\right)}^{-1}(\mu_{1}^{\left(l\right)}-\mu_{n}^{\left(l\right)})$. The distribution of the quadratic form can be approximated with a general $\chi^{2}$ distribution.

### 3.5. Choice of Weighting Parameters

Substituting (31) into the weighted sum of the information gains (35), it will be

(46) $$\pi =\arg\underset{\pi }{\inf}\sum_{n\in U}w_{n}\sum_{l\in L}\left[D_{KL}\left(r_{n}^{\left(l\right)}||r_{1}^{\left(l\right)}\right)+r_{n}^{\left(l\right)}D_{KL}\left(p_{n}^{\left(l\right)}||p_{1}^{\left(l\right)}\right)\right],$$

where the same weight $w_{n}$ is used for all l∈L. We revise the information gains as

(47) $$\pi =\arg\underset{\pi }{\inf}\sum_{l\in L}\sum_{n\in U}w_{n}^{\left(l\right)}\left[D_{KL}\left(r_{n}^{\left(l\right)}||r_{1}^{\left(l\right)}\right)+r_{n}^{\left(l\right)}D_{KL}\left(p_{n}^{\left(l\right)}||p_{1}^{\left(l\right)}\right)\right],$$

i.e., the different weight $w_{n}^{\left(l\right)}$ is used for different l∈L.

From the fused covariance $\Sigma^{\left(l\right)}$ in (43), the optimality in the mean square error sense is as follows [36]

(48) $$\underset{w_{n}^{\left(l\right)}}{\min}\mathrm{Trace}\left(\Sigma^{\left(l\right)}\right)=\underset{w_{n}^{\left(l\right)}}{\min}\mathrm{Trace}\left({\left(\sum_{n\in U}w_{n}^{\left(l\right)}{\left(\Sigma_{n}^{\left(l\right)}\right)}^{-1}\right)}^{-1}\right).$$

For each l∈L, a suboptimal solution is then given by

(49) $$w_{n}^{\left(l\right)}=\frac{1/\mathrm{Trace}\left(\Sigma_{n}^{\left(l\right)}\right)}{\sum_{m\in U}1/\mathrm{Trace}\left(\Sigma_{m}^{\left(l\right)}\right)}.$$

The suboptimal solution is calculated for each track l.

The proposed algorithm is summarized in Algorithm 1. The computational burden at the first sensor mainly includes two parts: (1) The first sensor computes the KLDs $D_{KL}(\pi_{n}||\pi_{1})$ in (31) for each n with $D_{KL}(r_{n}^{\left(l\right)}||r_{1}^{\left(l\right)})$ in (32) and $D_{KL}(p_{n}^{\left(l\right)}||p_{1}^{\left(l\right)})$ in (41); (2) The first sensor computes the fused parameters $\Sigma^{\left(l\right)}$ in (43) and $\mu^{\left(l\right)}$ in (44).
**Algorithm 1**: Multisensor Multitarget Tracking Against FDI Attacks**Input**: The prior density $\pi_{k-1}={\left\{\left(r_{k-1}^{\left(l\right)},p_{k-1}^{\left(l\right)}\right)\right\}}_{l\in L_{-}}$. **Output:** The posterior density $\pi_{k}={\left\{\left(r_{k}^{\left(l\right)},p_{k}^{\left(l\right)}\right)\right\}}_{l\in L}$.1. The first sensor receives the measurements $Z_{n,k}$ in (8) from the _*n*_th sensor (n=2,⋯,N). 2. The first sensor computes the predicted density ${\left\{\left(r_{k|k-1}^{\left(l\right)},p_{k|k-1}^{\left(l\right)}\right)\right\}}_{l\in L}$ in (15). 3. For each measurement
$Z_{n,k}$ (n=1,⋯,N), the first sensor computes the local updated density $\pi_{n,k}={\left\{\left(r_{n,k}^{\left(l\right)},p_{n,k}^{\left(l\right)}\right)\right\}}_{l\in L}$ in (25) with (19) and (20), where $p_{n,k}^{\left(l\right)}$ is approximated as a Gaussian density in (40). 4. The first sensor detects the FDI attacks with (30), where $D_{KL}(\pi_{n}||\pi_{1})$ is computed with (31)–(33) and (41). 5. The first sensor computes the fused density $\pi_{k}={\left\{\left(r_{k}^{\left(l\right)},p_{k}^{\left(l\right)}\right)\right\}}_{l\in L}$ with (37), (38), and (42)–(45).

## 4. Performance Evaluation

In this section, we present the performance evaluation of the proposed algorithm via simulation experiments.

### 4.1. Simulation Setup

The considered scenario is to track multiple targets that move inside the [−1000,1000]m×[−1000,1000]m surveillance area. The target’s state is represented by a 4-D vector, with 2-D position and 2-D velocity components. The survival probability of the target with state $x_{k}$ is set to a constant $p_{s}\left(x_{k}\right)=0.99$. The target dynamics of the survival targets are described by the constant velocity model in (1), with the sampling interval T=1(s) in (2) and (3). The process noise intensity in (3) is set to $q_{u}=1(m)$. The LMB density in (14) and (39) of the newly born targets includes 4 terms, which is denoted as $L_{k}=\{1,2,3,4\}$. Each term has the following form:

(50) $$\mu_{b}^{\left(1\right)}={\left(600,-10,-600,10\right)}^{T},\Sigma_{b}^{\left(1\right)}=100I_{4};$$

(51) $$\mu_{b}^{\left(2\right)}={\left(-800,10,-200,10\right)}^{T},\Sigma_{b}^{\left(2\right)}=100I_{4};$$

(52) $$\mu_{b}^{\left(3\right)}={\left(-400,10,-600,10\right)}^{T},\Sigma_{b}^{\left(3\right)}=100I_{4};$$

(53) $$\mu_{b}^{\left(4\right)}={\left(-200,10,800,-10\right)}^{T},\Sigma_{b}^{\left(4\right)}=100I_{4}.$$

The birth probability of each term in (14) is $r_{b}^{\left(l\right)}=0.03$, l=1,2,3,4.

**Figure 1:**
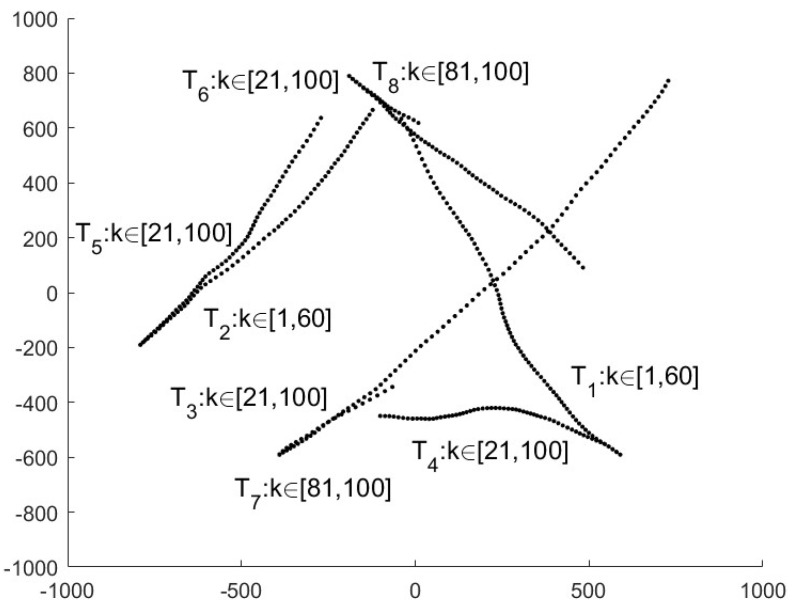
The starting and ending times of the trajectories of eight targets $T_{1},\cdots ,T_{8}$, e.g., $T_{2}\in [1,60]$ denote that the 2nd target appears at k=1 and disappears at k=60.

During the simulations, the total time steps are set to K=100. It is assumed that no target exists at the initial time k=0. Two new targets appear at time k=1, k=21, k=41 and k=81 respectively, and two existing targets disappear at time k=60. The starting and ending times of the trajectories of eight targets are depicted in Figure 1.

The surveillance area is monitored by a sensor network with N sensors that N=5,7,⋯,15 will be considered respectively. The detection probability of each sensor is set to a constant. $p_{d}(x_{k})=0.98$. The variance of measurement noise in (5) is set to

(54) $$R_{n,k}=\sigma_{v}^{2}I_{2},$$

where $\sigma_{v}^{2}=5$ m and $\sigma_{v}^{2}=10$ m will be considered later. The number of false alarms received by each sensor is distributed by a Poisson distribution with mean $\mu_{f}=10$. The spatial density κ(⋅) that describes each false alarm is a uniform distribution over the surveillance area.

We evaluate the tracking performance in terms of the optimal subpattern assignment (OSPA) metric [37]. Denote the actual multitarget state at time k as $X=\{x_{1},\cdots ,x_{n}\}$ and the estimated multitarget state as $Y=\{y_{1},\cdots ,y_{m}\}$ (we remove the time index k for briefly), where n and m are the number of actual and estimated targets, respectively. The OSPA metric is defined as

(55) $${\bar{d}}_{p}^{\left(c\right)}\left(X,Y\right)≜{\left(\frac{1}{n}\left(\underset{\pi \in \Pi_{n}}{\min}\sum_{i=1}^{m}d^{\left(c\right)}{\left(x_{i},y_{\pi (i)}\right)}^{p}+c^{p}(n-m)\right)\right)}^{\frac{1}{p}},$$

if m≤n, and ${\bar{d}}_{p}^{\left(c\right)}\left(X,Y\right)≜{\bar{d}}_{p}^{\left(c\right)}\left(Y,X\right)$ if _*m* > *n*_, where $\Pi_{n}$ is the set of permutations on {1,⋯,n}, $d^{\left(c\right)}(x,y)≜\min\left(c,d(x,y)\right)$ is the distance between x and y cut off at c>0, and 1≤p<+∞. The OSPA metric can capture the cardinality errors and state errors meaningfully. The parameters for the OSPA metric are set to p=1 and the cutoff parameter c=50 in the simulations.

### 4.2. Choice of Parameter _τ_

We consider that the FDI data $\vartheta_{n,k}$ in (6) has the form

(56) $$\vartheta_{n,k}=\rho \sigma_{v}^{2}{\left(1,1\right)}^{T},$$

where $\sigma_{v}^{2}$ is the measurement variance in (54), and different values of ρ will be considered in the experiments.

**Figure 2:**
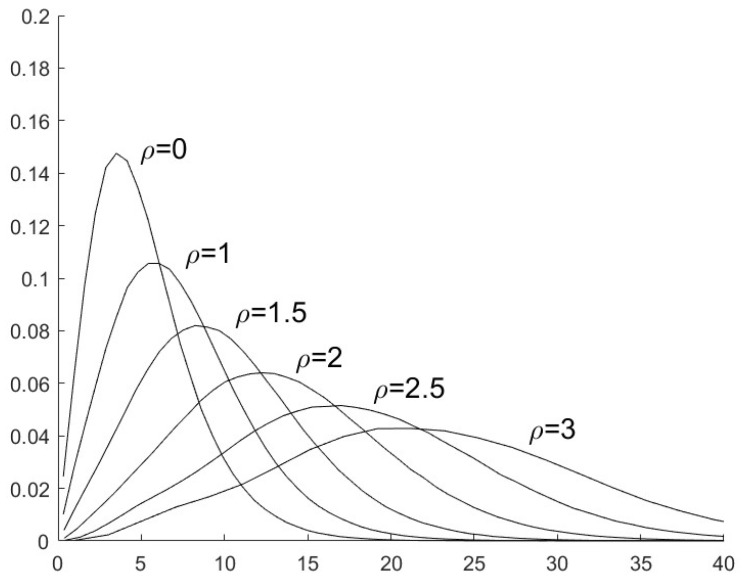
The distribution of $J(\pi_{n},\pi_{1})$ with different values of ρ and $\sigma_{v}^{2}=5$.

**Figure 3:**
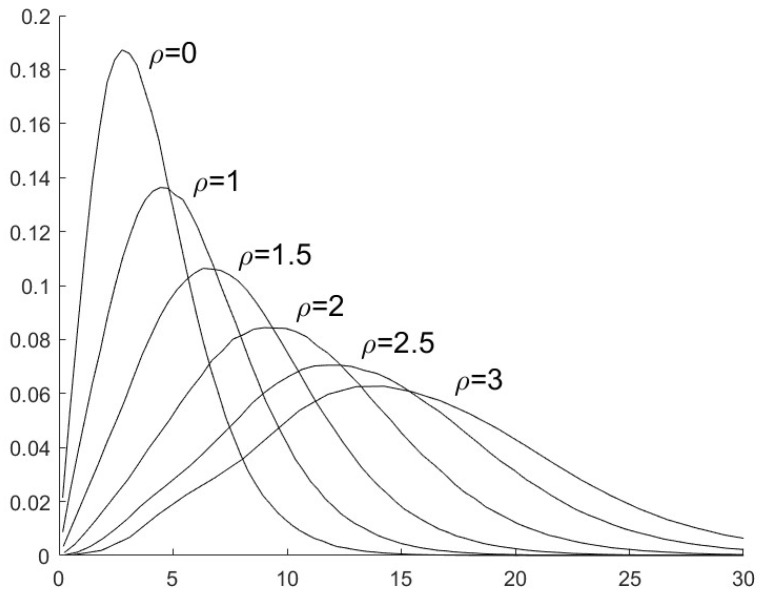
The distribution of $J(\pi_{n},\pi_{1})$ with different values of ρ and $\sigma_{v}^{2}=10$.

To choose the parameter _*τ*_ in (29), we first investigate the distributions of $J(\pi_{n},\pi_{1})$ when the sensor is secure or it is compromised by FDI attacks. With the simulation results, Figure 2 (where $\sigma_{v}^{2}=5$) presents the distributions of $J(\pi_{n},\pi_{1})$ when the sensor is secure, i.e., ρ=0, and when the sensor is compromised by FDI attacks with ρ=1,1.5,2,2.5,3. Figure 3 presents the distributions of $J(\pi_{n},\pi_{1})$ with $\sigma_{v}^{2}=10$ and ρ=0,1,1.5,2,2.5,3.

Let $J_{s}$ be the random variable that describes the distribution of $J(\pi_{n},\pi_{1})$ when the sensor is secure, and $J_{f}$ be the random variable that describes the distribution of $J(\pi_{n},\pi_{1})$ when the sensor is compromised by FDI attacks. We choose the parameter _*τ*_ as

(57) $$\tau =\arg\underset{\tau }{\min}\left\{\max\left(P\left(J_{s}>\tau \right),P\left(J_{f}<\tau \right)\right)\right\},$$

where $P\left(J_{s}>\tau \right)$ is the probability that the secure sensor is misjudged as a compromised sensor, and $P\left(J_{f}<\tau \right)$ is the probability that the compromised sensor is misjudged as a secure sensor. On the other hand, if we do not have the knowledge of $\vartheta_{n,k}$, we choose the parameter _*τ*_ as

(58) $$\tau =\underset{\tau }{\arg}\left\{P\left(J_{s}>\tau \right)=\tilde{P}\right\},$$

where $\tilde{P}$ is a suitable probability.

### 4.3. Experimental Results

We investigate and compare the following algorithms:

1. The proposed algorithm (PA1) where the parameter _*τ*_ is computed with (57).
2. The proposed algorithm (PA2) where the parameter _*τ*_ is computed with (58).
3. The comparison algorithm (CA1) that has the exact knowledge of the secure sensors and fuses the secure local estimates into a global estimate.
4. The comparison algorithm (CA2) that fuses all local estimates into a global estimate.
5. The comparison algorithm (CA3) that estimates the state only with the measurements from the first sensor.

**Figure 4:**
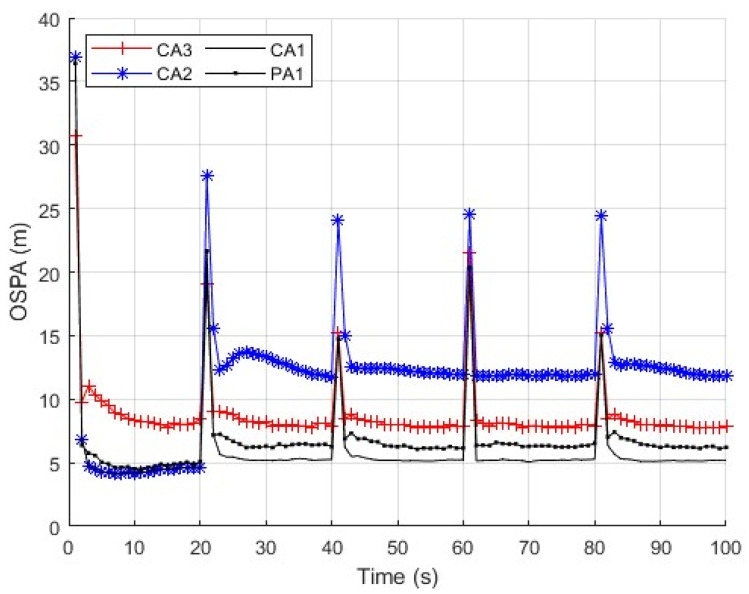
OSPA performance of the algorithms with ρ=2, $\sigma_{v}^{2}=10$ and N=5.

**Figure 5:**
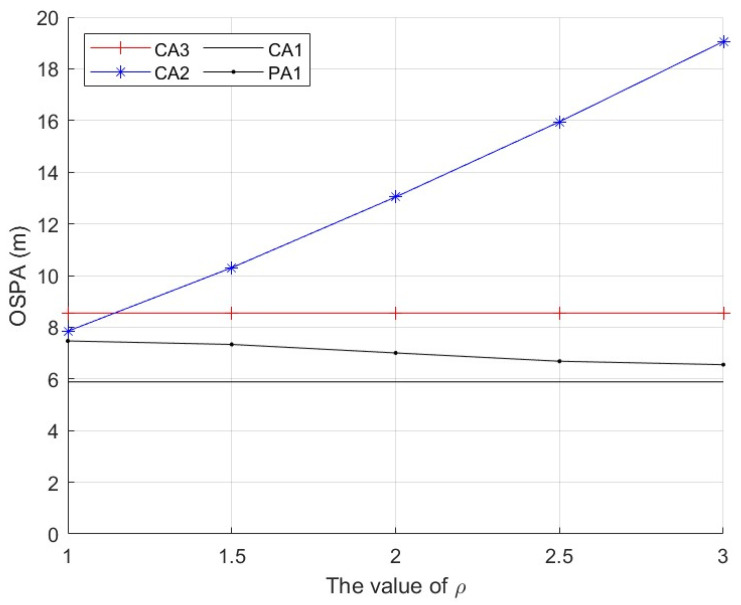
Time-average OSPA performance of the algorithms with different values of ρ, $\sigma_{v}^{2}=10$ and N=5.

**Figure 6:**
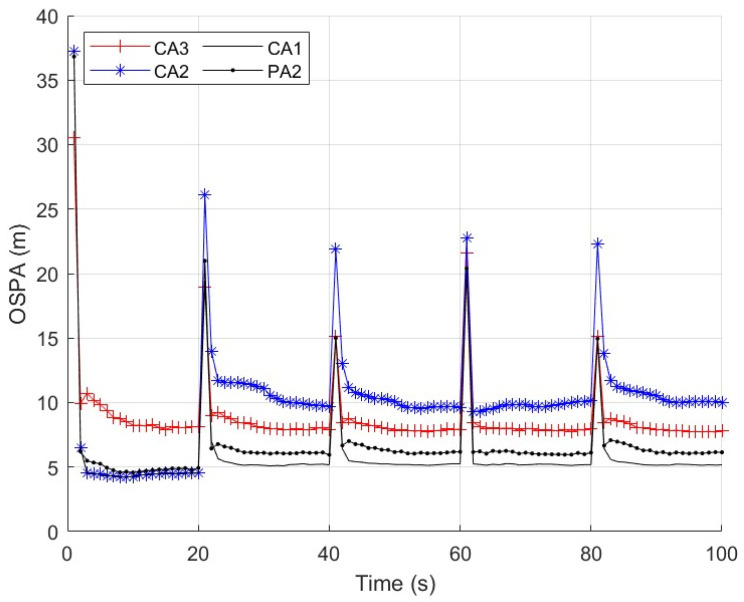
OSPA performance of the algorithms with ρ=|γ| where γ∼N(0,4), $\sigma_{v}^{2}=10$ and N=5.

First, we consider a sensor network with N=5 sensors. The first sensor is secure during whole the time interval. The second and third sensors are compromised by FDI attacks from time k=21 to k=60. The fourth and fifth sensors are comprised by FDI attacks from time k=61 to k=100. We repeat independent Monte Carlo runs to evaluate and compare the performance of these algorithms. The measurement noise variance is set to $\sigma_{v}^{2}=10$. Three cases of the parameter ρ are considered. Case 1: the parameter ρ in (56) is a constant ρ=2. The parameter _*τ*_ in (57) is then computed as τ=6.07 with (57) and the results in Figure 3. We present the average OSPA performance of the algorithms at each time step in Figure 4. Case 2: the parameter ρ in (56) is a constant as ρ=1,1.5,2,2.5,3. The parameter _*τ*_ in (30) is computed with (57) and the results in Figure 3. We present the time-average OSPA performance of the algorithms at these values of ρ in Figure 5. Case 3: ρ is not a constant and a new value of ρ is generated as ρ=|γ| where γ∼N(0,4) at the time k=21,31,⋯,91. The parameter _*τ*_ in (30) is computed with (58), where the probability $\tilde{P}$ is set to $\tilde{P}=0.16$ and the result _*τ*_ is τ=6.07. We present the average OSPA performance of the algorithms at each time step in Figure 6. From Figure 4, Figure 5 and Figure 6, the proposed algorithm can detect/defend the FDI attacks and provide the estimation performance that is close to the CA1, which has the exact knowledge of the secure sensors.

**Figure 7:**
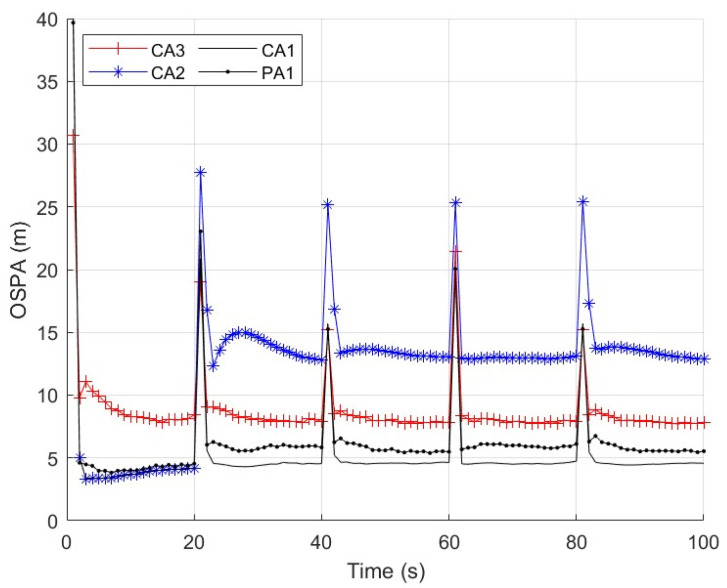
OSPA performance of the algorithms with ρ=2, $\sigma_{v}^{2}=10$ and N=9.

**Figure 8:**
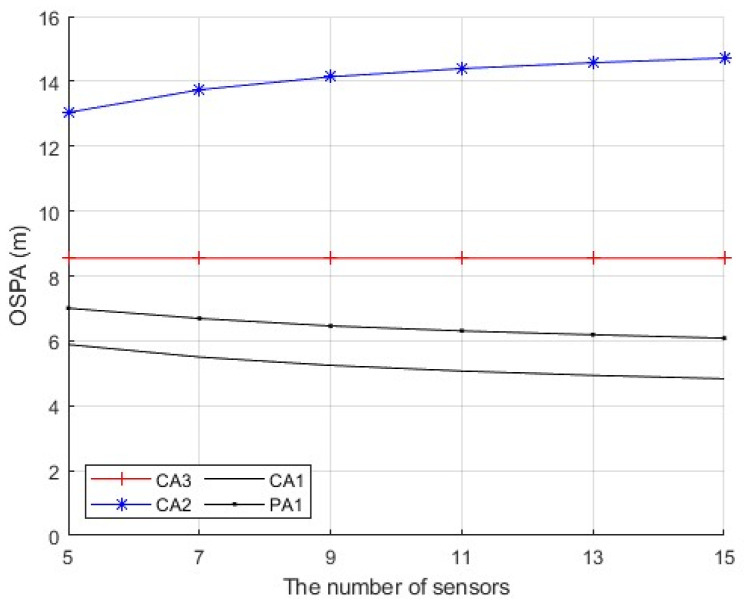
Time-average OSPA performance of the algorithms with ρ=2, $\sigma_{v}^{2}=10$ and different number N.

**Figure 9:**
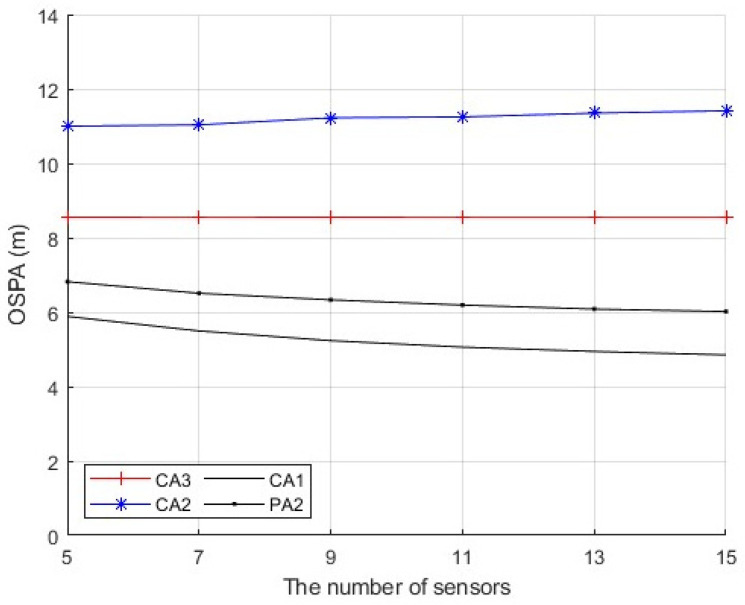
Time-average OSPA performance of the algorithms with ρ=|γ| where γ∼N(0,4), $\sigma_{v}^{2}=10$ and different number N of sensors.

Second, we consider a sensor network with N=5,7,⋯,15 sensors. The first sensor is secure during whole the time interval. The sensors n∈{2,⋯,(N+1)/2} are compromised by FDI attacks from time k=21 to k=60. The sensors n∈{(N+1)/2+1,⋯,N} are compromised by FDI attacks from time k=61 to k=100. The number of attacked sensors is (N−1)/2 at each time from k=21 to k=100. The measurement noise variance is set to $\sigma_{v}^{2}=10$. Figure 7 presents the average OSPA performance of the algorithms when the parameter ρ in (56) is a constant ρ=2 and the number of sensors is N=9. Figure 8 presents the time-average OSPA performance of the algorithms when the parameter ρ in (56) is a constant ρ=2 and the number of sensors is N=5,7,⋯,15. Figure 9 presents the time-average OSPA performance of the algorithms when the parameter ρ is generated as ρ=|γ| where γ∼N(0,4) at the time k=21,31,⋯,91, and the number of sensors is N=5,7,⋯,15. From Figure 7, Figure 8 and Figure 9, as the number N of sensors increases, the proposed algorithm can provide better estimation performance, while the performance of CA2 becomes worse.

## 5. Conclusions

We proposed a multisensor multitarget tracking algorithm that can detect the possible FDI attacks over the sensor networks. With a successful FDI attack, an adversary can manipulate the estimated values of the state variables and disrupt the system’s operation. A local estimate is first generated from the measurements of each sensor based on the LMB filter. A detection method for FDI attacks is then derived based on the KLD between LMB densities. Finally, the global estimate is produced by GCI fusion from all secure local estimates. To select the value of the threshold parameter, the statistical characteristics of the KLD are also analyzed when the sensor is secure or compromised by FDI attacks. A set of suitable weight parameters is selected for the information fusion of all secure local estimates. An efficient Gaussian implementation is also presented for the linear state-space model. Extensive experimental results illustrate that the proposed algorithm can efficiently detect/defend the FDI attacks and provide reliable tracking performance.

## Data Availability

The raw data supporting the conclusions of this article will be made available by the authors on request.

## Abbreviations

The following abbreviations are used in this manuscript:

CPHD	Cardinalized Probability Hypothesis Density
FDI	False Data Injection
GCI	Generalized Covariance Intersection
GLMB	Generalized Labeled Multi-Bernoulli
KLD	Kullback–Leibler Divergence
LMB	Labeled Multi-Bernoulli
MB	Multi-Bernoulli
OSPA	Optimal SubPattern Assignment
PHD	Probability Hypothesis Density
RFS	Random Finite Set
